# Multi-region social-emotional network underlying the effects of spine pinching therapy on empathy-like behavior in a rat model of autism spectrum disorder

**DOI:** 10.3389/fnins.2026.1948910

**Published:** 2026-09-29

**Authors:** Mian Li, Qing Tang, Wenzheng Tang, Shuhui Lin, Min Zhang, Xiaonan Guo, Yueyang Liu, Dong Lin, Lili Lin

**Affiliations:** 1 Fujian Academy of Chinese Medical Sciences, Fuzhou, Fujian, China; 2 College of Acupuncture, Fujian University of Traditional Chinese Medicine, Fuzhou, Fujian, China; 3 National Institute of TCM Constitution and Preventive Medicine, School of Chinese Medicine, Beijing University of Traditional Chinese Medicine, Beijing, China; 4 Rehabilitation Hospital Affiliated to Fujian University of Traditional Chinese Medicine, Fuzhou, Fujian, China

**Keywords:** autism, empathy ability, multi-brain region linkage, oxytocin, spine pinching

## Abstract

**Background:**

Spine pinching is a promising alternative for autism spectrum disorder (ASD), yet its effects on multi-region molecular coordination remain unclear. This study examined the effects of spine pinching on cellular Fos proto-oncoprotein (c-Fos) and Oxytocin Receptor (OXTR) expression in the prefrontal cortex, hypothalamus, and amygdala, and further analyzed expression patterns across these regions using profile analysis to explore the coordinated multi-region regulation underlying improvements in social and empathic deficits.

**Methods:**

An ASD rat model was established by valproic acid (VPA) induction. After behavioral testing, rats were divided into the model group (MG), spine pinching group (SPG), and blank control group (BC). SPG received a 15-day intervention from postnatal day 22. Then, open field, three-chamber social, and fear empathy tests were performed; brain tissues were collected for immunohistochemistry and western blotting, and profile analysis was conducted.

**Results:**

VPA-induced ASD rats showed developmental and ASD-like behavioral deficits. MG rats exhibited social and empathy deficits and reduced c-Fos and OXTR levels in the prefrontal cortex, hypothalamus, and amygdala versus BC. Spine pinching improved these ASD-like behavioral deficits. Immunohistochemistry revealed that c-Fos and OXTR expression increased significantly in all three brain regions after spine pinching. Western blot analysis further supported these findings, showing significant upregulation in most brain regions and consistent upward trends in the remaining regions. Profile analysis revealed parallel expression profiles across regions, suggesting coordinated multi-region regulation.

**Conclusion:**

Spine pinching effectively improves core ASD-like behavioral deficits in ASD rats, potentially through coordinated regulation of the multi-regional social-emotional network.

## 1 Introduction

Autism spectrum disorder (ASD), a neurodevelopmental condition with complex etiology, is clinically characterized by impairments in social interaction, narrow interests, and repetitive and stereotyped behaviors (Lord et al., 2018). In recent years, the prevalence of ASD has demonstrated a marked upward trend globally. According to data from the Global Burden of Disease study published in *The Lancet Psychiatry* in 2025, more than 61.8 million people worldwide were living with ASD by 2021, corresponding to a global prevalence of approximately 1 in 127 individuals (Global, 2025). The 2024 Report on the Development of Autism Education and Rehabilitation Industry in China reports a prevalence of approximately 0.7% among children aged 6–12 years, from which it is estimated that the number of autistic children aged 0–14 years may exceed 2 million (Wucailu Autism Research Institute, 2024).

Impaired empathic ability represents one of the core deficits of ASD (Baron-Cohen and Wheelwright, 2004; Harmsen, 2019; Schnitzler and Fuchs, 2024). This deficit leads to multifaceted impairments in social cognitive function, including difficulty understanding others’ emotions, an inability to accurately interpret social situations, and challenges in developing appropriate social behaviors. In neurobiological research, the discovery of the Mirror Neuron System (MNS) has provided a crucial theoretical foundation for understanding the neural basis of empathy and related social cognitive functions. MNS activation enables individuals to comprehend others’ behavioral intentions and emotional states through a process of “embodied simulation,” thereby establishing the neurobiological substrate for empathic communication (Rizzolatti and Craighero, 2004; Fan et al., 2011). Clinical evidence demonstrates that when completing tasks involving imitation and observation of emotional expressions, patients with ASD exhibit significantly lower activation levels in brain regions associated with the MNS compared to typically developing children (Dapretto et al., 2006). Studies have shown that rodent models can exhibit social and empathic-like behaviors, including observational fear and social interaction (Pereira et al., 2012; Zhang et al., 2024; Tang et al., 2025). These behaviors similarly depend on a neural circuit comprising brain regions such as the prefrontal cortex, anterior cingulate cortex, insula, hypothalamus, and amygdala (Gunaydin and Deisseroth, 2014). These brain regions demonstrate substantial anatomical and functional homology and overlap with the human MNS (Molenberghs et al., 2012) and function synergistically with the MNS during empathic processing.

Spine pinching therapy, a traditional Chinese external treatment modality, was first documented in *Handbook of Prescriptions for Emergencies* by Ge Hong of the Eastern Jin Dynasty. In this ancient text, the procedure is described as a continuous pinching and lifting of the skin and subcutaneous tissue along the paravertebral region, moving from the Changqiang acupoint (GV1) to the Dazhui acupoint (GV14), with the manipulation repeated until the therapeutic effect is achieved (Figure 1A). In traditional Chinese medicine, the spine region is considered to be associated with the Governor Vessel, and the Governor Vessel has a close relationship with brain function. Spine pinching therapy, by stimulating the spine region, purportedly activates the Qi of the Governor Vessel to ascend and nourish the brain, thereby achieving effects such as supplementing marrow to benefit the brain, and opening orifices to awaken the spirit. Clinically, it has been applied in the treatment of mental and emotional disorders (Zhang, 2013). In recent years, the therapeutic efficacy of spine pinching therapy has gained increasing recognition, and mechanistic studies into its effects are progressively being conducted and deepened (Li et al., 2017). Our research group’s preliminary studies have confirmed that spine pinching therapy can effectively improve social interaction and cognitive behavior in ASD model rats (Wu et al., 2020; Gong et al., 2023; Su et al., 2024; Tang et al., 2024). Furthermore, we have found that tuina intervention at the Dazhui acupoint significantly elevates serum oxytocin expression in ASD model rats (Wu, 2025).

**FIGURE 1:**
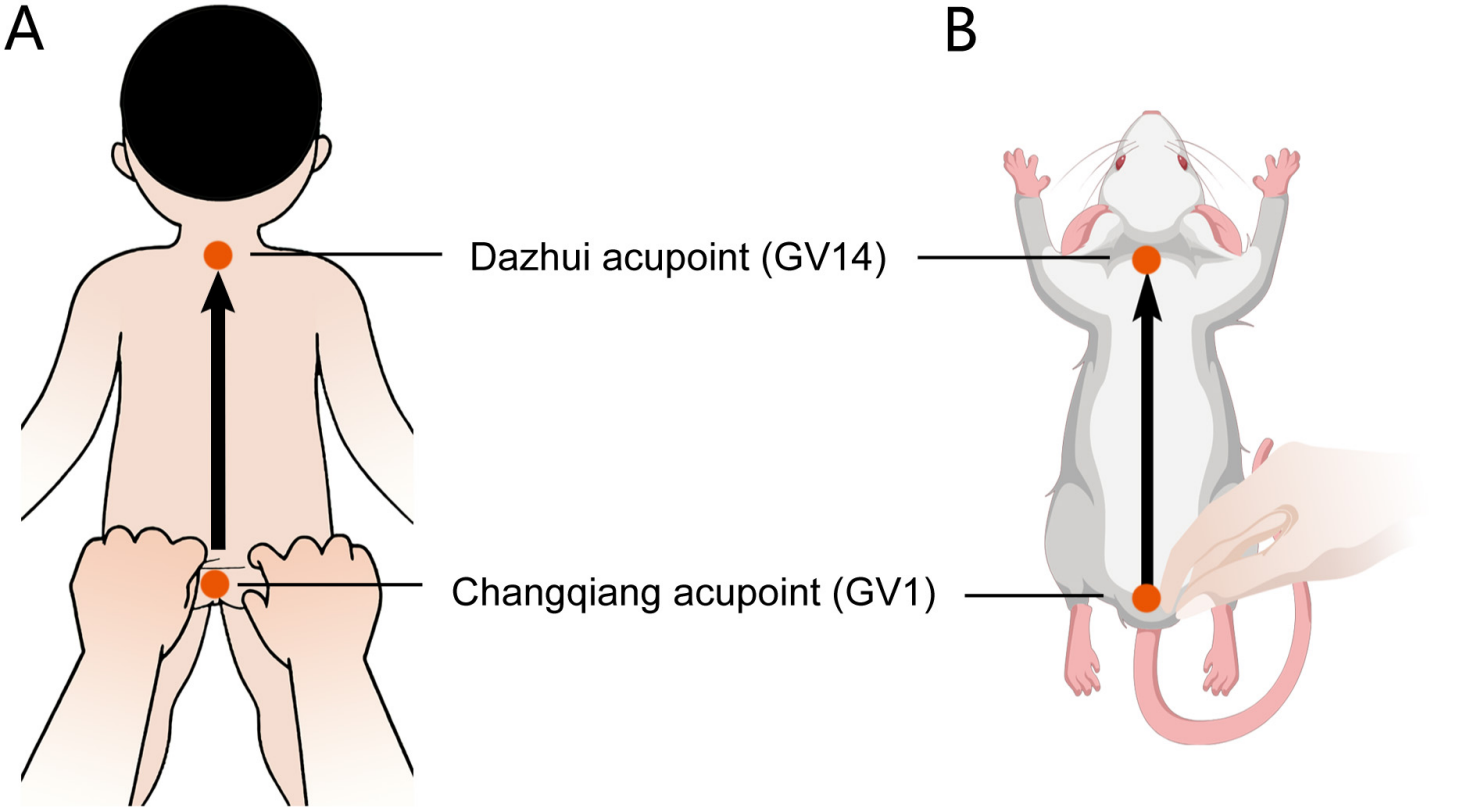
Schematic diagram of spine pinching manipulation. **(A)** Manipulation schematic for children. **(B)** Manipulation schematic for rats. The mouse elements in this Figure were created using (https://BioGDP.com) (Jiang et al., 2025).

Unlike previous studies that focused on a single behavioral endpoint or molecular changes in a single brain region, the present study employed a multi-level design combining developmental phenotyping, multi-dimensional behavioral tests, region-specific immunohistochemical and western blot analyses, and profile analysis, aiming to more comprehensively characterize the coordinated changes across multiple social-emotional brain regions underlying the improvement of empathic ability by spine pinching therapy in ASD. To this end, building upon our team’s prior research, this study aims to investigate the effects of spine pinching therapy on the levels of cellular Fos proto-oncoprotein (c-Fos) and Oxytocin Receptor (OXTR) in multiple brain regions associated with social empathic behavior in ASD model rats, and to characterize the patterns of multi-region coordination using profile analysis.

## 2 Materials and methods

### 2.1 Animals

Wistar rats (males: 220 ± 20 g; females: 200 ± 20 g, 2 months old) were obtained from Shanghai SLAC Laboratory Animal Co., Ltd. Rats were housed in the Animal Experiment Center of Fujian University of Traditional Chinese Medicine under specific pathogen-free (SPF) conditions, with constant temperature and humidity, and a 12-h light/dark cycle. Animals were maintained in a controlled environment with ad libitum access to food and water. A total of 36 rats (18 males and 18 females) were used in this study. Animals were acclimated to the housing conditions for at least 7 days prior to any experimental procedures.

### 2.2 Model establishment

Adult rats were paired overnight at a 1:1 male-to-female ratio. On the following morning, cages were examined for vaginal plugs; females with plugs were designated as embryonic day 0.5 (E0.5). At E12.5, one group of pregnant dams received an intraperitoneal injection of Valproic Acid (VPA) solution at 600 mg/kg, and their pups were designated as the VPA-exposed group; another group of pregnant dams received an equivalent volume of saline at the same time point, and their pups were regarded as the control group. The day of birth was designated as postnatal day (PN) 1.

Developmental assessments, including body weight measurement and eye-opening test, and developmental behavioral tests, including negative geotaxis test, swimming test, and hot plate pain test, were conducted at various time points during the pups’ growth. To evaluate the success of VPA-induced model establishment, a pup was considered to have successfully modeled ASD if it exhibited significant differences from control animals (*P* < 0.05) in at least 4 of the above developmental assessments and developmental behavioral tests.

A total of 8 VPA-treated dams and 10 saline-treated dams were used. Litter sizes ranged from 8 to 12 pups in the VPA-exposed group and 10 to 14 in the control group. In the present study, 82 pups were obtained from the 8 VPA-treated dams. After systematic developmental screening, 58 pups met the modeling criteria, yielding a success rate of 70.7%, which is consistent with previously reported rates for the VPA-induced ASD model (Schneider and Przewłocki, 2005; Zhang et al., 2026). From these 58 model-positive pups, 24 were randomly selected using a random number table and assigned to the model group (MG, *n* = 12) and the spine pinching group (SPG, *n* = 12). Simultaneously, 12 pups from saline-treated dams were randomly selected as the blank control group (BC, *n* = 12). The remaining animals were excluded from subsequent statistical analysis. Additionally, 36 age-matched untreated rats were co-housed with the experimental rats and served only as observation subjects for the three groups in the fear empathy test.

To minimize potential litter effects, pups from different litters were selected whenever possible, with a maximum of two pups from the same litter assigned to the same experimental group. Sex ratios were balanced across groups, and no significant differences were detected by chi-square test (*P* > 0.05). No animals or data points were excluded from the final analysis.

### 2.3 Experimental design and spine pinching therapy intervention

The SPG was subjected to the intervention starting from postnatal day 22 (PN22). The intervention was performed once daily, with 21 repetitions per session, for 15 consecutive days. The intensity of the manipulation was adjusted according to the tolerance of the rats (i.e., the rats did not vocalize and remained relatively calm), following the procedures described in our previous studies (Wu et al., 2020; Gong et al., 2023; Su et al., 2024; Tang et al., 2024). Specifically, the operator wore cotton gloves and gently pressed the rat’s head with the left hand to stabilize it, while the right hand pinched the skin on both sides of the spine using the thumb pad in opposition to the index and middle finger pads, advancing from the Changqiang acupoint (GV1) along the midline toward the head, ending at the Dazhui acupoint (GV14) (Figure 1B). Every three pinches, the skin was lifted once, which was counted as one repetition. Prior to the formal experiment, the operator practiced repeatedly to ensure the manipulation was consistent and smooth. All procedures were performed by the same experimenter. Rats in the BC and MG received sham grasping (i.e., manual restraint and skin contact without spine pinching) to control for non-specific stress effects.

Following the spine pinching intervention, open field tests, three-chamber social test, and fear empathy test were conducted. Rats were anesthetized and perfused for tissue collection within 5 min after the completion of the fear empathy test. Behavioral tests were video-recorded by a technician who was not involved in animal grouping. All behavioral videos were analyzed by investigators blinded to the experimental groups. Image acquisition and quantification of immunohistochemical staining, as well as densitometric analysis of western blot bands, were also performed by investigators blinded to group allocation. No animals or data points were excluded from the final analysis.

### 2.4 Developmental assessments and developmental behavioral tests

Body Weight: The body weight of each pup in all groups was measured and recorded on PN7, PN14, and PN21.

Eye-Opening Test: The eye-opening status of each pup in all groups was observed and recorded on PN12, PN13, PN14, PN15, and PN16.

Negative Geotaxis Test: This test was performed on PN7, PN8, PN9, and PN10. During testing, the pup was placed on a smooth slope inclined at 25°, with its head positioned facing downward. The time required for the pup to complete a 180° turn was recorded.

Swimming Test: This test was conducted on PN9, PN11, PN13, and PN15. During testing, each pup was gently placed into a water tank maintained at a constant temperature of 28°C. Within 10 s, the water level relative to the pup’s head position was observed, and a score was recorded.

Hot Plate Pain Test: The determination of the thermal pain threshold was performed using a PL-200 plantar thermal stimulator. Testing was scheduled on PN9, PN11, PN13, and PN15. Prior to testing, the glass plate was cleaned with alcohol. Subsequently, the pup was transferred to a dry container lined with wood shavings for acclimation. After the pup’s condition stabilized, its left hind paw pad was aligned with the infrared light source, and the heating program was initiated, with the timer starting simultaneously. The timer was stopped immediately when the animal exhibited a paw withdrawal response due to the thermal stimulus, and the recorded duration was used as the pain threshold indicator. Each pup was tested three times, with a 5-min interval between trials. The mean value of the three measurements was calculated as the final result. To minimize experimental error, all tests were conducted during a fixed time period each day. Furthermore, this test was scheduled as the final assessment on the day of developmental behavioral testing to prevent any interference from the plantar thermal stimulation on the results of other experiments.

### 2.5 Behavioral tests

#### 2.5.1 Open field test

Rats were individually placed in a clean open-field apparatus (72 × 72 × 42 cm), followed by a 5-min testing period. Between trials, the apparatus was cleaned with 75% ethanol to eliminate odor cues. The SuperMaze system was used to automatically record the total distance traveled and the number of entries into the central zone within a 5-min period. The experimenter simultaneously counted the number of grooming and rearing behaviors.

#### 2.5.2 Three-chamber social test

The three-chamber social test apparatus was divided by transparent partitions into three equal compartments (each measuring 30 × 40 × 30 cm), with a semicircular passage (radius: 60 mm) in the center of each partition. A metal wire cage (18 cm in height, 8 cm in diameter) was placed in each of the two lateral compartments. After a 5-min habituation period in the central compartment, the test rat was removed, and an unfamiliar rat was placed into the wire cage in the left compartment, while the wire cage in the right compartment remained empty. Subsequently, the test rat was reintroduced into the central area, and its activity was monitored for 10 min. The time spent in each lateral compartment was recorded. The social index was calculated as the ratio of time spent exploring the side containing the unfamiliar rat to the total time spent exploring both sides. Between trials, the testing apparatus was cleaned with 75% ethanol to remove odor cues.

#### 2.5.3 Fear empathy test

The conditioned fear empathy experimental apparatus used in this study comprised a soundproof and light-proof chamber (80 × 80 × 80 cm), which effectively minimized interference from the external environment, a conditioned fear chamber (30 × 30 × 44 cm), and a conditioned stimulus controller along with a video acquisition system. The entire system was controlled by the VisuFcs software, a scene-based fear analysis system.

On the first day of the test, three groups of observer rats received electric foot shocks to enhance their negative experiential exposure. Observer rats were placed in a cage on the electrified side and subjected to a single electric foot shock (1 mA, 2 s) with a 4 s inter-shock interval, repeated ten times. 24 h after the single shock session, the observer rats were placed in the shock zone for a 5-min acclimatization period and then transferred to the adjacent observation zone to familiarize themselves with the environment.

In addition, normal rats of similar age were selected as demonstrator rats. These demonstrator rats were placed in the shock zone and subjected to electric foot shocks (0.4 mA, 2 s) with a 12 s inter-shock interval, repeated 25 times over 5 min. The duration of freezing behavior exhibited by the observer rats during the 5-min observation period was recorded. This parameter was used to reflect the observer’s perception of fear-related emotional information and served as the basis for determining whether the observer demonstrated empathy-like behavior toward the demonstrator. The schematic diagram of the test is shown in Figure 2.

**FIGURE 2:**
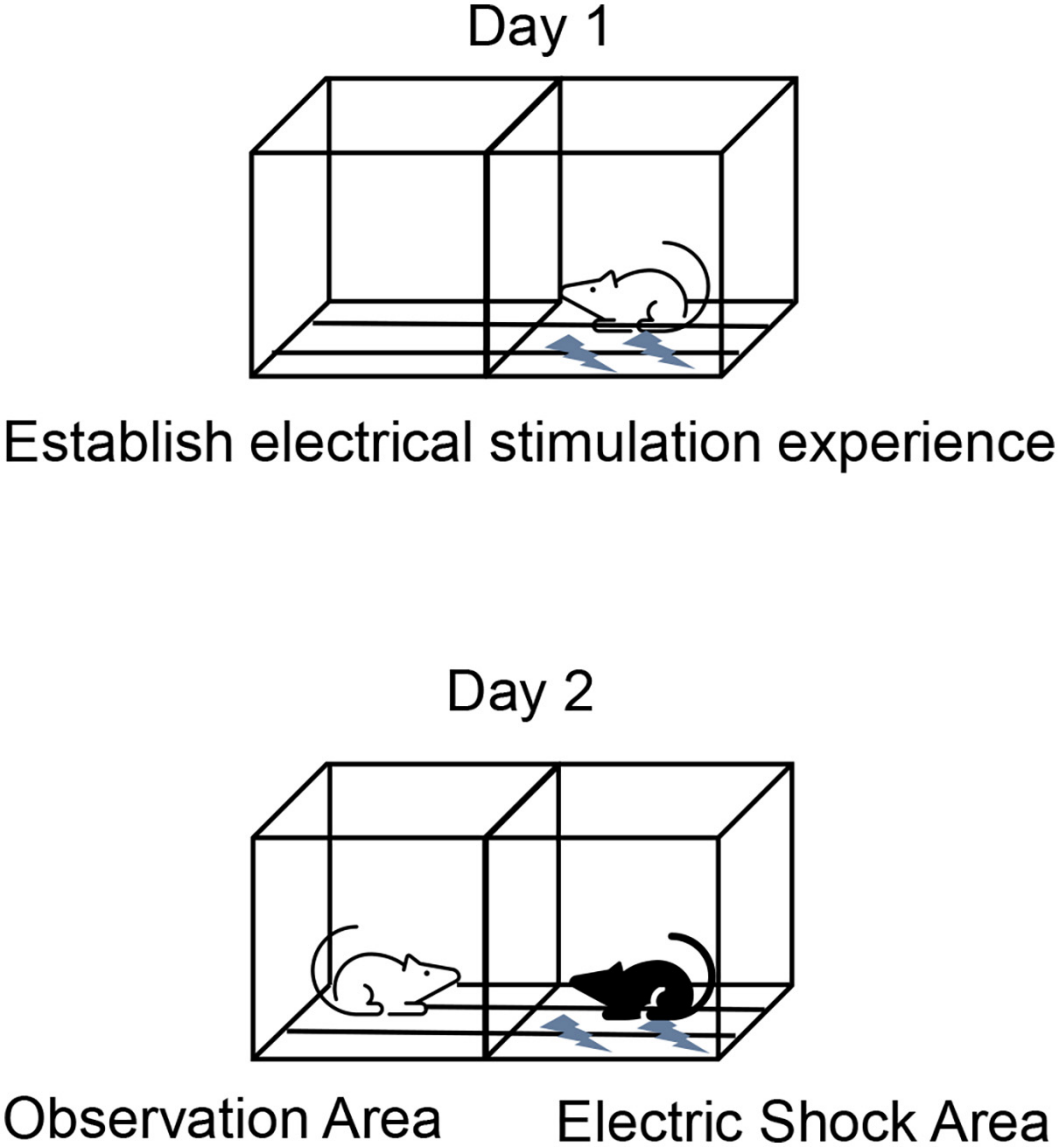
Schematic diagram of the fear empathy test apparatus and procedure. Day 1: Observer rats received electric foot shocks (1 mA, 2 s, 10 times, 4 s inter-shock interval) in the shock zone. Day 2: Observer rats were allowed to acclimatize in the shock zone for 5 min and then moved to the adjacent observation zone; demonstrator rats received electric foot shocks (0.4 mA, 2 s, 25 times, 12 s inter-shock interval) in the shock zone over 5 min. The freezing behavior of observer rats was recorded during the 5-min observation period.

### 2.6 Immunohistochemical detection of mean optical density in various brain regions

Brain tissue was immersed in 4% paraformaldehyde for 24 h, followed by gradient alcohol dehydration and fixation, and subsequently embedded in paraffin. The tissue blocks were sectioned into 4 μm slices, baked in a 60°C oven for 1 h, and then deparaffinized and rehydrated. The tissue sections were immersed in citrate antigen retrieval solution for 10 min and then naturally cooled to room temperature, followed by thorough washing with PBS. Subsequently, the sections were treated with 3% endogenous peroxidase blocker for 10 min and thoroughly washed with PBS. A non-specific staining blocker was then applied for 30 min at 37°C. Primary antibodies were applied and incubated overnight at 4°C. The following day, the tissue slides were placed in a 37°C incubator for 30 min, followed by thorough washing with PBS. Mouse/rabbit IgG polymer was applied and incubated for 30 min at 37 °C, followed by thorough washing with PBS. Finally, streptavidin-peroxidase was added and incubated for 10 min at room temperature, followed by thorough rinsing with PBS. DAB and hematoxylin staining times were determined through optimization, and staining was performed accordingly. The sections were observed and photographed under a microscope, and images were subsequently analyzed.

Images were captured at ×400 magnification and analyzed using ×400 panoramic views. Quantification was performed using ImageJ software. Briefly, images were converted to 8-bit grayscale, and a fixed gray threshold was applied to eliminate background non-specific staining. Based on the rat brain atlas of Paxinos and Watson (Paxinos and Watson, 2014), the following brain regions and their subregions were selected for analysis: prefrontal cortex (medial prefrontal cortex from bregma +3.20 mm to +2.70 mm), hypothalamus (paraventricular nucleus and surrounding regions from bregma −1.80 mm to −2.12 mm), and amygdala (basolateral and central nuclei from bregma −2.12 mm to −3.60 mm). For each region of interest, the integrated optical density (IOD) and area were measured, and the mean optical density (MOD) was calculated as MOD = IOD/Area. For each animal, one coronal section containing the maximal cross-sectional area of each target brain region was selected for quantification.

### 2.7 Detection of protein expression in various brain regions by western blotting

For each 100 mg of tissue, 1 mL of pre-cooled protein lysis buffer was added, and the homogenate was incubated on ice for 30 min. The mixture was then centrifuged at 12,000 rpm for 10 min at 4 °C, and the supernatant was collected for subsequent protein concentration determination. Marker and equal amounts of protein samples were loaded into the lanes. Electrophoresis was performed at a constant voltage of 20 V for 10 min, followed by 80 V for 40 min, and then 120 V for an additional 60 min. The PVDF membrane was activated in methanol. The transfer sandwich was placed into the transfer tank, and wet transfer was performed at 400 mA for 38 min. The PVDF membrane was rinsed with TBST and blocked for 1 h at room temperature. After discarding the blocking solution, primary antibodies were applied and incubated overnight on a shaker at 4 °C for no less than 12 h. The membrane was then removed and thoroughly rinsed with TBST buffer. Subsequently, secondary antibodies were applied and incubated on a shaker for 1 h at room temperature, followed by thorough washing with TBST. In a darkroom, equal volumes of Solution A and Solution B from the ECL chemiluminescent substrate kit were mixed and applied dropwise onto the membrane surface, followed by chemiluminescent development and image acquisition. The gray values of the bands were quantified using ImageJ software, and the relative protein expression levels were determined.

### 2.8 Statistical methods

Statistical analysis was performed using SPSS 27.0 and GraphPad Prism 10. A *P* value of less than 0.05 was considered statistically significant. Normally distributed data are presented as mean ± SD, otherwise as median (P25, P75).

For developmental data and brain region-wise protein expression, repeated-measures two-way ANOVA was used with group as the between-subjects factor and time or brain region as the within-subjects factor, respectively, followed by Holm-Šidák’s post-hoc test. For single-time-point behavioral comparisons among three groups, one-way ANOVA was used, followed by Šidák’s post-hoc test, or the Kruskal–Wallis test when appropriate. Profile analysis was performed using the Repeated Measures module of SPSS to evaluate coordinated multi-region regulation.

Profile analysis was performed within the framework of a general linear model for repeated measures, with “group” (BC, MG, SPG) as the between-subjects factor and “brain region” (prefrontal cortex, hypothalamus, amygdala) as the within-subjects factor. The MOD or relative protein expression levels of each target (c-Fos and OXTR) across the three brain regions were entered as dependent variables. Three sequential tests were conducted to characterize the pattern of coordinated inter-regional regulation:

Test of parallelism: This test examined the interaction between group and brain region. A *P* > 0.05 indicates that the expression profiles across the three brain regions are parallel among groups, suggesting that the intervention effect is directionally consistent across regions. A *P* < 0.05 would suggest region-specific effects of the intervention.

Test of levels: Under the condition that the profiles are parallel, this test further examined whether the profiles are at the same overall level, i.e., comparing the global mean expression across the three brain regions among groups. A *P* < 0.05 indicates a systematic difference in overall expression levels between groups.

Test of flatness: Using data from all groups combined, this test examined whether the mean expression levels differed across the three brain regions, indicating whether the baseline expression abundance of a given protein is balanced across regions. A *P* < 0.05 suggests that the protein exhibits inherently higher expression in certain brain regions.

## 3 Results

### 3.1 The effects of prenatal exposure to VPA on the development of rats

In body weight measurements (Figure 3A), compared with the control group, the body weights of the VPA-exposed group on PN7, PN14, and PN21 were significantly lower. In the eye-opening test (Figure 3B), compared with the control group, the eye-opening scores of the VPA-exposed group on PN15 and 16 were significantly reduced. These results suggest delayed growth and development in the VPA-exposed group. In the negative geotaxis test (Figure 3C), compared with the control group, the VPA-exposed group required significantly more time to complete the task on PN7, PN8, PN9, and PN10, indicating impairment of vestibular function in the VPA-exposed group. In the swimming test (Figure 3D), compared with the control group, the VPA-exposed group exhibited significantly lower test scores on PN9, PN11, PN13, and PN15, suggesting decreased motor coordination and endurance in the VPA-exposed group. In the hot plate pain test (Figure 3E), compared with the control group, the VPA-exposed group showed increased hot plate pain thresholds on PN9, PN11, PN13, and PN15, indicating reduced pain sensitivity in the VPA-exposed group. These findings are consistent with the established developmental and sensorimotor phenotypes of the VPA-induced ASD model (Schneider and Przewłocki, 2005; Zhang et al., 2026).

**FIGURE 3:**
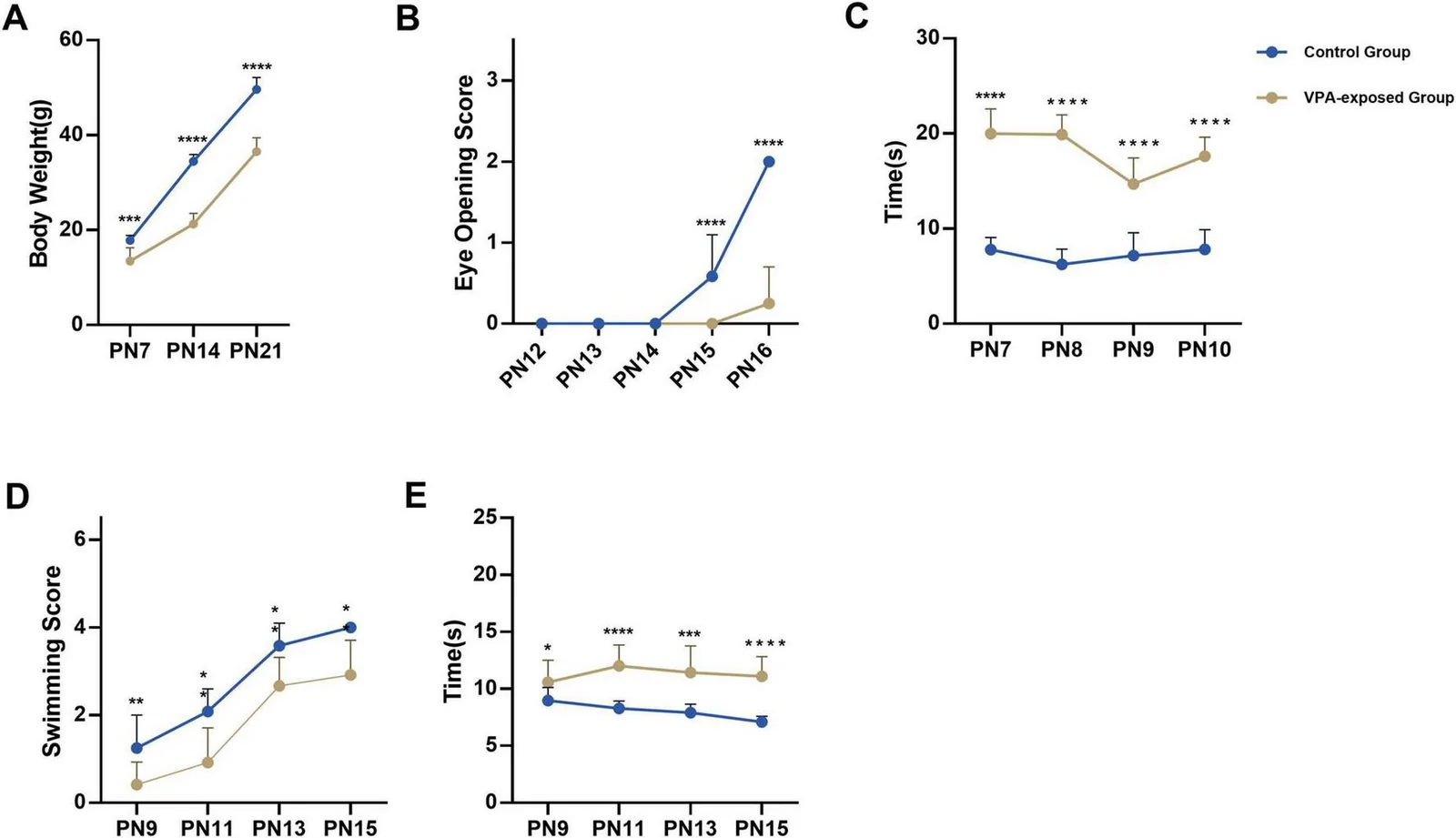
Effects of prenatal VPA exposure on developmental and behavioral parameters in rats. Comparisons of body weight, eye-opening test, negative geotaxis test, swimming test, and hot plate test between control group and VPA-exposed group rats. **(A)** Body weight of rats at different postnatal days. Data are expressed as mean ± SD, *n* = 12, two-way ANOVA. **(B)** Eye-opening scores at different postnatal days. Data are expressed as median (P25, P75), *n* = 12, two-way ANOVA. **(C)** Latency to complete the negative geotaxis test at different postnatal days. Data are expressed as mean ± SD, *n* = 12, two-way ANOVA. **(D)** Swimming scores at different postnatal days. Data are expressed as median (P25, P75), *n* = 12, two-way ANOVA. **(E)** Hot plate pain thresholds at different postnatal days. Data are expressed as mean ± SD, *n* = 12, two-way ANOVA. Statistical significance in the graphs is indicated as *P < 0.05, *P < 0.05, **P < 0.01, ***P < 0.001, **** *P* < 0.0001.

### 3.2 The effect of spine pinching on the behavior of ASD rats

In the open field test (Figures 4A–D), compared with the BC, the MG exhibited significantly decreased total distance traveled, number of entries into the central area, and number of rearing, alongside a significantly increased number of grooming. Compared with the MG, the SPG demonstrated significantly increased total distance traveled, number of entries into the central area, and number of rearing, with a significantly reduced number of grooming. These findings suggest that spine pinching intervention can alleviate anxiety-like behaviors and enhance exploratory drive in ASD model rats.

**FIGURE 4:**
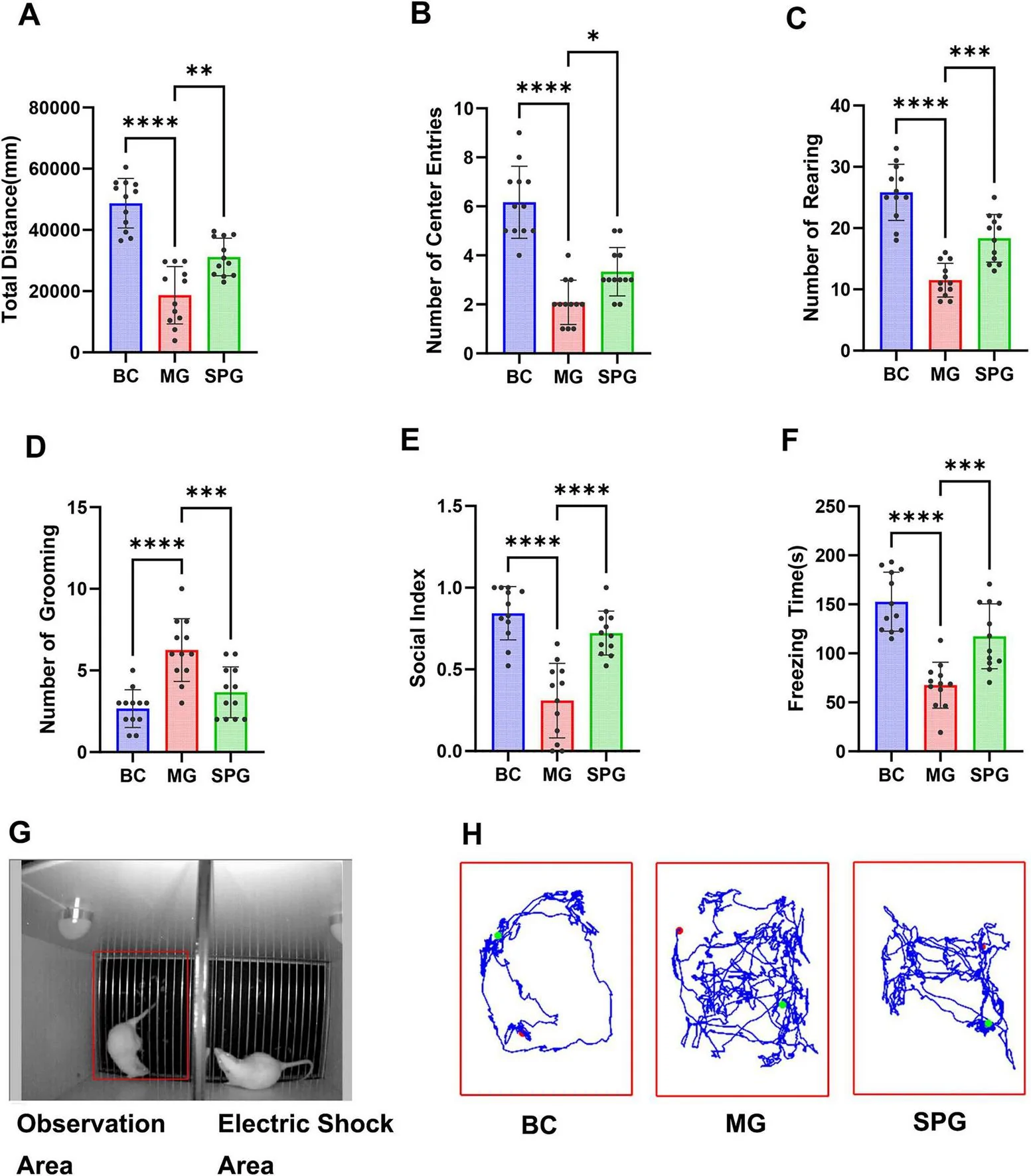
Effects of spine pinching intervention on behavior in ASD model rats. Comparison of open field test, three-chamber social test, and fear empathy test among three groups. **(A)** Total distance traveled in the open field test, *n* = 12, one-way ANOVA. **(B)** Number of entries into the central area in the open field test, *n* = 12, one-way ANOVA. **(C)** Number of rearing in the open field test, *n* = 12, one-way ANOVA. **(D)** Number of grooming in the open field test, *n* = 12, one-way ANOVA. **(E)** Social index in the three-chamber social test, *n* = 12, one-way ANOVA. **(F)** Freezing duration in the fear empathy test, *n* = 12, one-way ANOVA. **(G)** Representative photographs of the fear empathy test apparatus. **(H)** Representative trajectory plots of observer rats in the observation zone during the fear empathy test. All data are expressed as mean ± SD. Statistical significance in the graphs is indicated as *P < 0.05, **P < 0.01, ***P < 0.001, **** *P* < 0.0001.

In the three-chamber social test (Figure 4E), the social index was significantly decreased in the MG compared with the BC. Relative to the MG, the social index was significantly increased in the SPG, indicating that spine pinching intervention can ameliorate social deficits and enhance social initiative in ASD model rats.

Deficits in empathic ability are considered one of the core features of social dysfunction in ASD. The duration of freezing behavior displayed by observer rats upon witnessing conspecifics receiving foot shocks was used as an indicator of their ability to perceive and share the emotional state of fear in the observed rats (Jeon et al., 2010). In the fear empathy test (Figures 4F–H), the duration of freezing behavior was significantly decreased in the MG compared with the BC. Conversely, compared with the MG, the freezing duration was significantly increased in the SPG. These results suggest that model rats exhibit impaired emotional empathy, and that spine pinching intervention can, to some extent, enhance the empathic ability of ASD model rats.

### 3.3 Effects of spine pinching on c-fos and OXTR in different brain regions of ASD model rats

To investigate the effects of spine pinching on the activation of different brain regions in a rat model of ASD, the expression of c-Fos was examined in the prefrontal cortex, hypothalamus, and amygdala. As depicted in Figure 5, immunohistochemical analysis revealed that the MOD of c-Fos in these three brain regions was significantly lower in the MG compared to the BC. In contrast, compared with the MG, the SPG exhibited a significantly higher MOD of c-Fos in all three brain regions. In western blot analysis (Figure 6), the relative expression levels of c-Fos in all three brain regions of MG rats were significantly decreased compared with the BC. Compared with the MG, the c-Fos levels in the prefrontal cortex and amygdala of SPG rats were significantly increased. Although the hypothalamus showed a consistent upward trend, the difference was not statistically significant.

**FIGURE 5:**
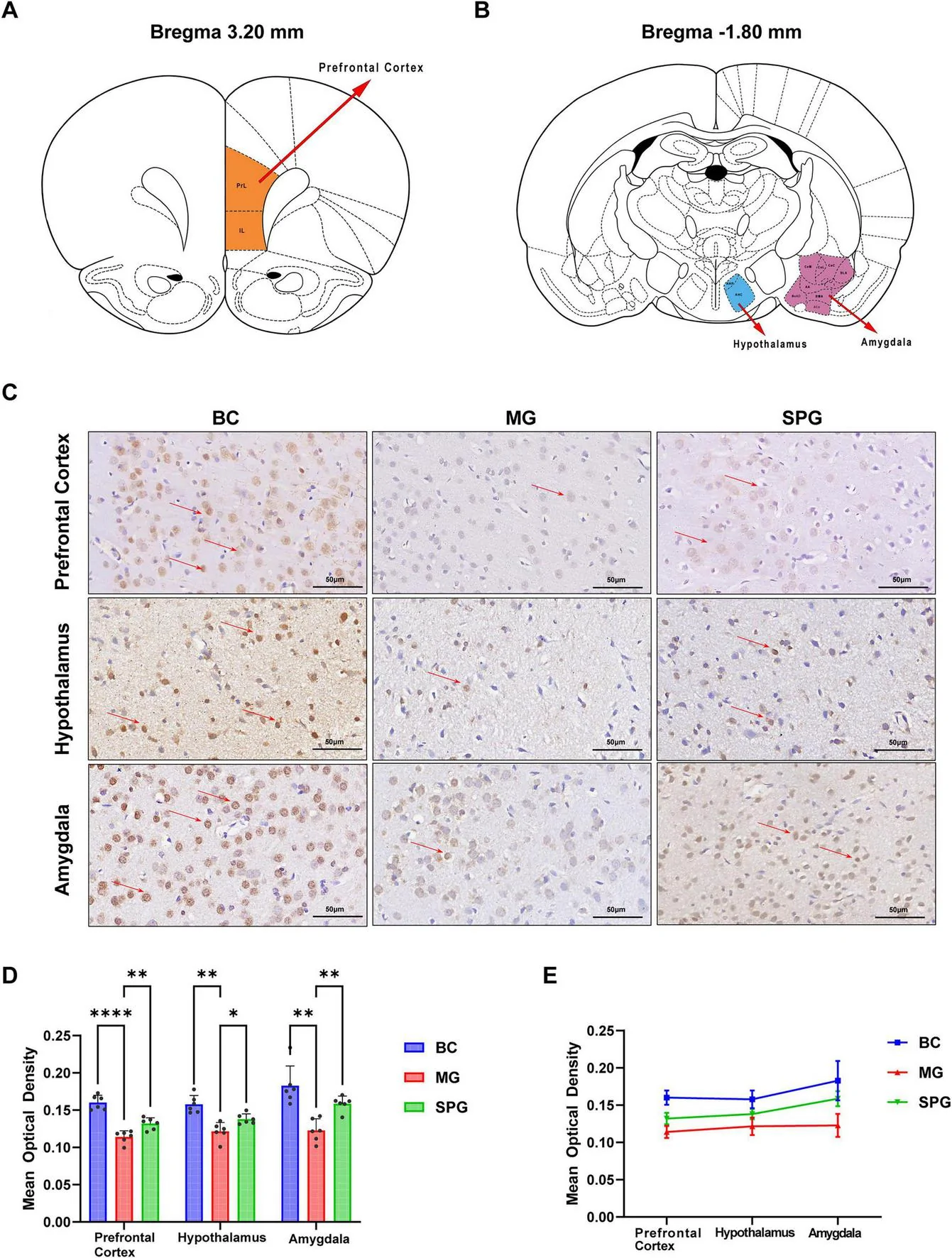
Effects of spine pinching on c-Fos expression in different brain regions of ASD model rats. Comparison of c-Fos expression in the prefrontal cortex, hypothalamus, and amygdala among the three groups. **(A)** Schematic diagram of a coronal rat brain section showing the anatomical location of the prefrontal cortex at approximately bregma +3.20 mm. **(B)** Schematic diagram of a coronal rat brain section showing the anatomical locations of the hypothalamus and amygdala at approximately bregma –1.80 mm. **(C)** Immunohistochemical images of c-Fos expression in different brain regions of the three groups. **(D)** Comparison of MOD values of c-Fos expression in different brain regions of the three groups, two-way ANOVA. **(E)** Profile analysis of MOD values of c-Fos expression in different brain regions of the three groups. For each animal, one coronal section containing the largest cross-sectional area of each target brain region was selected for MOD quantification. All data are expressed as mean ± SD (*n* = 6 per group). Statistical significance in the graphs is indicated as *P < 0.05, **P < 0.01, ****P < 0.0001.

**FIGURE 6:**
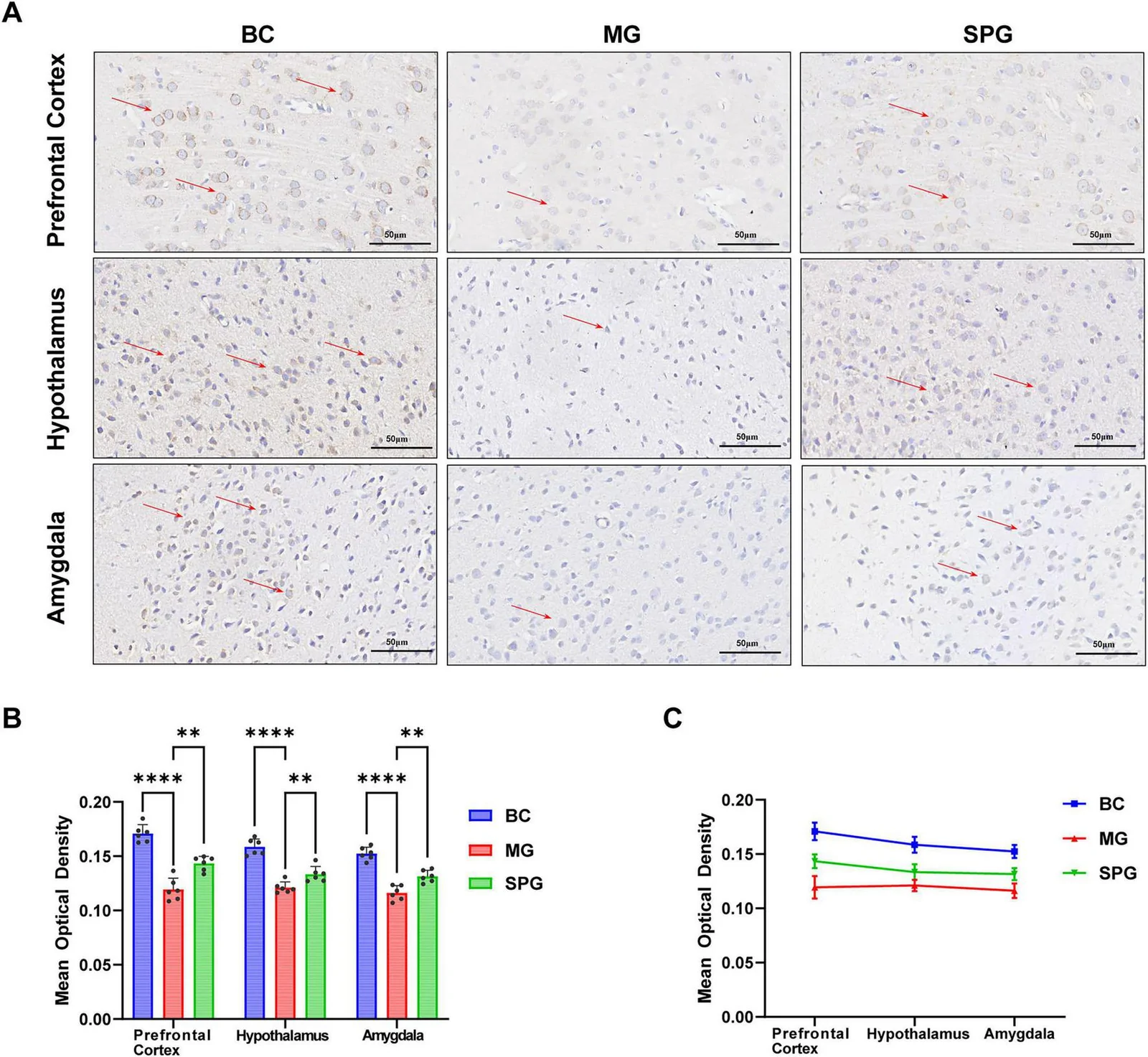
Effects of spine pinching on c-Fos protein expression in different brain regions of ASD model rats. Comparison of relative c-Fos protein expression levels in the prefrontal cortex, hypothalamus, and amygdala among the three groups. **(A)** Western blot bands of relative c-Fos protein expression in different brain regions of the three groups. **(B)** Comparison of relative c-Fos protein expression levels in different brain regions of the three groups, two-way ANOVA. **(C)** Profile analysis of relative c-Fos protein expression levels in different brain regions of the three groups. For western blotting, each biological sample was loaded in duplicate, and the gray values were averaged from three exposures per membrane for each animal. Data are expressed as mean ± SD (*n* = 6 per group). Statistical significance in the graphs is indicated as ns *P* > 0.05, *P < 0.05, **P < 0.01, ***P < 0.001.

Immunohistochemical analysis (Figure 7) revealed that the MOD of OXTR in all three brain regions was significantly lower in the MG than in the BC, while it was significantly higher in the SPG compared to the MG. In western blot analysis (Figure 8), the relative expression levels of OXTR in all three brain regions of MG rats were significantly decreased compared with the BC. Compared with the MG, the OXTR levels in the prefrontal cortex and hypothalamus of SPG rats were significantly increased, whereas in the amygdala, a similar upward trend was observed, but it was not statistically significant.

**FIGURE 7:**
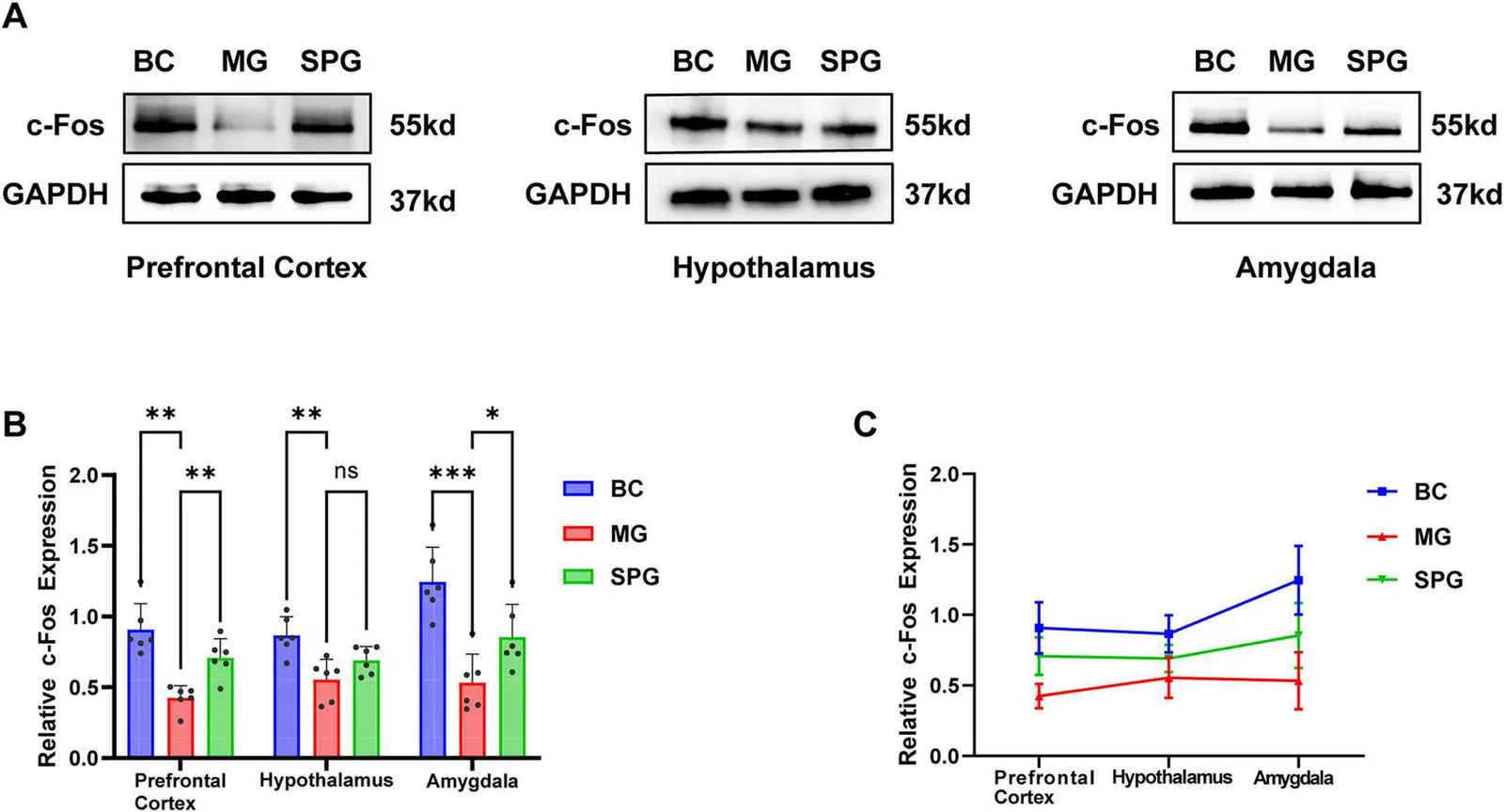
Effects of spine pinching on OXTR expression in different brain regions of ASD model rats. Comparison of OXTR expression in the prefrontal cortex, hypothalamus, and amygdala among the three groups. The schematic diagrams showing the anatomical locations of the prefrontal cortex, hypothalamus, and amygdala are presented in Figures 5A and B. **(A)** Immunohistochemical images of OXTR expression in different brain regions of the three groups. **(B)** Comparison of MOD values of OXTR expression in different brain regions of the three groups, two-way ANOVA. **(C)** Profile analysis of MOD values of OXTR expression in different brain regions of the three groups. For each animal, one coronal section containing the largest cross-sectional area of each target brain region was selected for MOD quantification. All data are expressed as mean ± SD (*n* = 6 per group). Statistical significance in the graphs is indicated as **P < 0.01, ****P < 0.0001.

**FIGURE 8:**
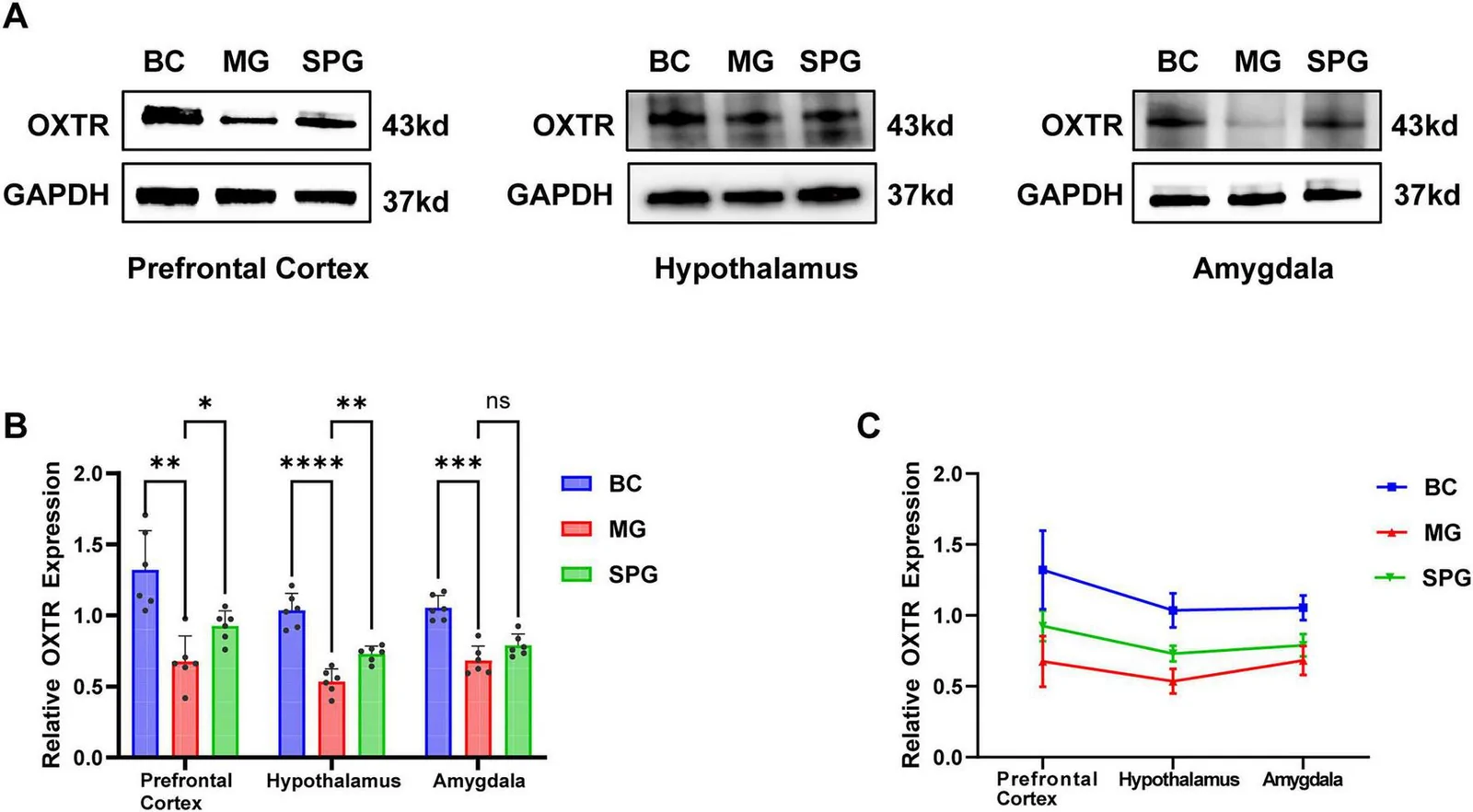
Effects of spine pinching on OXTR protein expression in different brain regions of ASD model rats Comparison of relative OXTR protein expression levels in the prefrontal cortex, hypothalamus, and amygdala among the three groups. **(A)** Western blot bands of relative OXTR protein expression in different brain regions of the three groups. **(B)** Comparison of relative OXTR protein expression levels in different brain regions of the three groups, two-way ANOVA. **(C)** Profile analysis of relative OXTR protein expression levels in different brain regions of the three groups. For western blotting, each biological sample was loaded in duplicate, and the gray values were averaged from three exposures per membrane for each animal. Data are expressed as mean ± SD (*n* = 6 per group). Statistical significance in the graphs is indicated as ns *P* > 0.05, *P < 0.05, **P < 0.01, ***P < 0.001, ****P < 0.0001.

### 3.4 Spine pinching intervention in ASD model rats induces multi-brain region synergistic effects

Profile analysis was used to evaluate the coordinated expression patterns of c-Fos and OXTR across the prefrontal cortex, hypothalamus, and amygdala among the three groups of rats. Using profile analysis, the test of parallelism revealed no significant differences in the expression trends of c-Fos and OXTR in the prefrontal cortex, hypothalamus, and amygdala among the three groups. The test of levels indicated significant differences in the overall mean levels of c-Fos and OXTR across the three brain regions among the three groups. The test of flatness suggested that the overall expression levels of c-Fos and OXTR differed significantly across the three brain regions, with c-Fos showing the highest expression in the amygdala and OXTR exhibiting the highest expression in the prefrontal cortex (Figures 5E, 6C, 7C, 8C). These findings suggest that spine pinching may improve empathic ability in ASD through coordinated regulation across multiple brain regions.

## 4 Discussion

Using a multi-level design integrating developmental phenotyping, behavioral assays, region-specific molecular measurements, and profile analysis, this study investigated the coordinated multi-region regulation induced by spine pinching in improving empathic ability in ASD model rats. In the human brain, the MNS is widely activated during tasks involving action intention processing, emotional resonance, and social cognition, constituting an important neural foundation for empathic ability (Flournoy et al., 2016). Rodent studies have indicated that the neural mechanisms of empathy may depend on a distributed brain network functioning synergistically with the MNS. For instance, when a rat observes another rat receiving an electrical shock, it exhibits freezing behavior. Concurrently, corresponding neuronal activity can be detected in brain regions such as the cingulate cortex, which is closely associated with cognitive and emotional regulation (Carrillo et al., 2019). Further research has demonstrated that empathic ability is not governed by a single brain region; rather, dysfunction in the functional coordination among multiple brain regions can lead to deficits in empathy (Chen et al., 2021; Ukezono and Takano, 2021).

The prefrontal cortex, hypothalamus, and amygdala were selected as target regions because they represent key nodes of the social-emotional network and functionally interact with the mirror neuron system. During empathic processing, the prefrontal cortex is responsible for cognitive integration (Mohapatra and Wagner, 2023), the amygdala participates in emotional evaluation (Bachevalier and Loveland, 2006; Adolphs, 2010), and the hypothalamus regulates physiological stress responses (Burkett et al., 2016; Pisansky et al., 2017; Rogers-Carter et al., 2018; Daviu et al., 2020). These three regions coordinate their functions and together contribute to the formation of empathic responses. Clinical studies have shown that functional connectivity between the prefrontal cortex and amygdala is significantly reduced in patients with ASD (Ibrahim et al., 2019), and abnormal activation patterns in the prefrontal cortex have been observed during social cognitive tasks (Kennedy and Courchesne, 2008; Gotts et al., 2012). Animal studies have similarly demonstrated that abnormalities in these brain regions are closely associated with social deficits in ASD model rats (Sacai et al., 2020; Higuchi et al., 2025). Therefore, examining these regions allows us to assess whether spine pinching can coordinately regulate this social-emotional network.

In the present study, spine pinching intervention was initiated at PN22 (roughly corresponding to early childhood in humans) and continued until PN36, a period that represents a critical window for neurodevelopmental plasticity in rats, characterized by active synaptic pruning and circuit refinement (Penzes et al., 2011; Chen et al., 2025). Our previous study found that spine pinching has therapeutic effects in ASD model rats at PN23, PN37, and PN51, with greater improvements observed at PN23 and PN37 than at PN51, suggesting that the therapeutic efficacy of spine pinching is more pronounced during early developmental stages (Tang et al., 2024). Early intervention is considered crucial for the prognosis of ASD, and thus this developmental stage was selected to allow observation of the effects of spine pinching.

Individuals with ASD commonly exhibit deficits in empathic ability, characterized by difficulty in recognizing and understanding the emotional states of others, as well as an inability to infer their behaviors and intentions (McKenzie et al., 2022). With ongoing research, the MNS, which is intimately linked to empathic ability, has been confirmed to be abnormal in the brains of individuals with ASD. Deficits in imitative ability in patients with ASD are associated with neuronal dysfunction in the amygdala and the orbitofrontal cortex (OFC) (Zheng et al., 2017). Based on our previous findings, we suggest that spine pinching may improve ASD-like behaviors by regulating social-emotional brain regions. Since ASD core symptoms are unlikely to arise from a single brain region, the MNS theory highlights the necessity of multi-region coordination in social cognition, which offers a valuable neurobiological framework for understanding the holistic effects of spine pinching.

c-Fos protein is a phosphorylated protein encoded by the immediate-early gene c-Fos. As a key marker of neuronal activation, it can be rapidly induced by various stressors and reflects the activation status of neurons in corresponding brain regions (Herrera and Robertson, 1996). c-Fos can serve as a functional tracing tool for investigating the neural pathways and their spatiotemporal characteristics activated by specific stimuli (Dong et al., 2025). Because the regional distribution, temporal dynamics, and expression intensity of c-Fos vary with different types of stimuli, this method has become an important approach for studying neural functional states and circuits. For example, Yang et al. found that prenatal stress exposure led to learning and memory impairment in offspring rats, accompanied by elevated c-Fos expression in the hippocampus and hypothalamus (Yang et al., 2021). Other researchers have also used c-Fos expression in pain-related brain regions to identify key areas involved in the therapeutic effects of manipulation (Huang et al., 2025). Thus, as a commonly used immediate-early gene protein, c-Fos expression can serve as one of the morphological indicators of neuronal activation in response to specific behaviors.

Oxytocin is a neuropeptide synthesized in the paraventricular nucleus and supraoptic nucleus of the hypothalamus (Ross and Young, 2009). Current research indicates that oxytocin is closely associated with social behavior (Jones et al., 2017), and abnormalities in oxytocin may lead to deficits in empathy, social dysfunction, and anxiety-like behaviors (Pantouli et al., 2024). Yawning contagion is considered a manifestation of social empathy, and researchers have found a positive correlation between plasma oxytocin levels and yawning contagion scores in children with ASD (Mariscal et al., 2019). In rodent studies, genetic models with OXTR gene knockout or functional defects consistently exhibit typical ASD-like behaviors (Pobbe et al., 2012). Wei et al. demonstrated that oxytocin intervention in ASD model rats activates the ERK/MAPK signaling pathway, thereby improving social deficits (Wei et al., 2020). Tactile stimulation, such as massage, has been shown to increase oxytocin levels in individuals with ASD and activate neural circuits associated with the reward system (Chen et al., 2020). Moreover, it has been proposed that stimulation of cutaneous CT fibers through tuina may trigger an increase in oxytocin levels, leading to pleasant sensations and ultimately alleviating social deficits in patients (Zhi et al., 2024). Our previous work similarly found that tuina intervention significantly increased serum oxytocin expression in ASD model rats (Wu, 2025). Therefore, we selected c-Fos and OXTR as the research indicators.

We examined the expression of c-Fos and OXTR in the prefrontal cortex, amygdala, and hypothalamus. Immunohistochemical and western blot analyses consistently showed that the expression of c-Fos and OXTR was downregulated in the prefrontal cortex, amygdala, and hypothalamus of MG rats, suggesting that neuronal activation levels and oxytocin signaling in these brain regions, which are closely associated with emotion and social cognition, might be relatively low in ASD model rats. In most region-specific comparisons, spine pinching intervention significantly upregulated both proteins, whereas the non-significant regions, namely the hypothalamic c-Fos and amygdalar OXTR, also showed a consistent upward trend. Notably, c-Fos protein typically reaches its peak expression at 30 to 60 min after stimulation. In the present study, tissue samples were collected within 5 min after the fear empathy test. Therefore, the c-Fos expression detected in this experiment primarily reflects the neuronal activation induced by the experimental procedures, including the fear-empathy task itself, rather than the genuine basal level of neuronal activity. The downregulation of OXTR in the prefrontal cortex, hypothalamus, and amygdala of MG rats may reflect insufficient oxytocin signaling in empathy-related brain regions, which could be an important molecular basis for their social reward deficits and empathy impairments. As a form of superficial mechanical stimulation, spine pinching involves rhythmic and gentle lifting and pinching of the back skin. This may activate somatosensory nerve fibers in the back skin that are associated with pleasant touch, which then project upward to a multi-regional social-emotional network. Through this pathway, spine pinching may positively regulate the oxytocin signaling system across these brain regions, thereby improving social and empathic deficits.

Further analysis employed contour analysis to conduct multivariate testing on protein expression data across the three brain regions. The test of parallelism revealed no significant differences among groups in the trends of c-Fos and OXTR changes across the three brain regions, indicating that spine pinching induced a synergistic and directionally consistent regulation of these molecules within a brain network encompassing the prefrontal cortex, hypothalamus, and amygdala. The test of levels was significant, revealing systematic shifts in the overall expression levels among the three groups. The model state led to overall suppression of this synergistic network, whereas spine pinching exhibited a global upregulating and restorative effect. The test of flatness further demonstrated the expression characteristics of each protein within each brain region, showing inherent differences in baseline expression levels of c-Fos and OXTR across the three regions. Among these, c-Fos expression was most prominent in the amygdala, which may reflect its role as a key hub for emotion and stress response, where the neuronal activity marker c-Fos exhibits relatively high basal activity. OXTR expression was highest in the prefrontal cortex, consistent with its functional role as a higher-order integration center where social behavior, decision-making, and emotional regulation rely heavily on oxytocin signaling. These inherent expression differences were maintained across groups, further corroborating the conclusion of the test of parallelism, namely that spine pinching intervention did not alter the functional proportional distribution of different molecules across different brain regions. These findings suggest that the therapeutic effects of spine pinching on ASD-related social and empathic deficits may be mediated through coordinated multi-region regulation, rather than through isolated actions on a single brain area.

The lack of statistical significance for hypothalamic c-Fos and amygdalar OXTR in western blot analysis does not undermine our overall conclusion. These proteins showed consistent upward trends in these regions, and profile analysis confirmed parallel multi-region regulation. The significant increases detected by immunohistochemistry in the same regions further corroborate the biological effects. This discrepancy may reflect inherent differences in sensitivity between the two techniques. Western blot measures total protein from heterogeneous tissue homogenates, which may underestimate local changes in region-specific subnuclei, whereas immunohistochemistry offers higher spatial resolution. Therefore, these results collectively support a synergistic multi-region regulatory mechanism of spine pinching. The regional differences in the magnitude of its effects warrant further investigation with larger sample sizes or fine-grained subregional dissections to enable comprehensive statistical detection in western blot analyses.

The present study has several limitations. First, we only examined c-Fos and OXTR, providing only a preliminary understanding of multi-region regulation; future studies using *in vivo* multichannel electrophysiology are needed to verify causal connectivity among brain regions. Second, although baseline activity and anxiety-like behavior were assessed by the open field test, the fear-empathy test may still be influenced by individual differences in sensitivity to social cues, and future investigations should incorporate more refined behavioral analyses to control for such confounders. Third, although sex ratios were balanced across groups, the sample size did not allow for sex-stratified analyses. Future studies are needed to investigate potential sex-specific effects of spine pinching in ASD models.

In summary, starting from the MNS-related social-emotional network, this study found that spine pinching applied to the back skin may activate somatosensory afferent pathways, upregulate the expression of c-Fos and OXTR in the prefrontal cortex, amygdala, and hypothalamus, and regulate the coordinated activity patterns among these brain regions, thereby achieving a holistic regulation of the MNS-related multi-regional social-emotional network. This integration of multi-region synergistic effects may serve as the potential basis for the simultaneous improvement of anxiety-like behaviors, social deficits, and empathy impairments in ASD model rats following spine pinching.

## 5 Conclusion

Spine pinching therapy effectively ameliorates core behavioral deficits, including social dysfunction and impaired empathic ability, in a VPA-induced rat model of ASD. This therapeutic effect may be closely associated with the concurrent modulation of key protein expression, such as c-Fos and OXTR, in the prefrontal cortex, hypothalamus, and amygdala. The present study provides preliminary experimental evidence supporting the hypothesis that spine pinching therapy ameliorates empathic deficits in ASD by synergistically regulating the functional connectivity patterns among multiple brain regions involved in social and empathic behaviors.

## Data Availability

The original contributions presented in the study are included in the article/supplementary material, further inquiries can be directed to the corresponding authors.
